# A Novel Ratiometric Fluorescent Probe for Mercury (II) ions and Application in Bio-imaging

**DOI:** 10.3390/molecules24122268

**Published:** 2019-06-18

**Authors:** Qianmiao Gao, Yang Jiao, Cheng He, Chunying Duan

**Affiliations:** 1 State Key Laboratory of Fine Chemicals, Dalian University of Technology, Dalian 116024, China; gaoqianmiao@outlook.com (Q.G); hecheng@dlut.edu.cn (C.H.); cyduan@dlut.edu.cn (C.D.); 2 School of Chemical Engineering, Dalian University of Technology, Dalian 116024, China

**Keywords:** fluorescent probe, Hg^2+^ ions, phenylimidazole

## Abstract

Since the accumulation of mercury (II) ions in the environment and ecosystem causes serious problems to environment and disease, the recognition of Hg^2+^ ions and its bio-imaging is of high importance. In sight of the advantages of fluorescence probes, a new probe (**PMH**) was facilely synthesized by incorporating phenylimidazole fluorophore and 3-methyl-2- benzothiazolinone hydrazone hydrochloride monohydrate. The **PMH** probe exhibited a ratiometric response for Hg^2+^ ions with fluorescence intensity increasing at 520 nm and decreasing at 445 nm simultaneously. The **PMH** probe interacted with Hg^2+^ ions in seconds with high optical stability and showed good selectivity over other metal ions. In addition, the probe has excellent biocompatibility and imaging performance in cells and zebrafish.

## 1. Introduction

Mercury is one of the most toxic elements widely existing in water and soil which brings serious threat to the environment and health along with the excessive utilizations in industry and agriculture [1,2,3,4,5,6]. The toxicity of Hg^2+^ ions is persistent, refractory, highly biodegradable, which is mainly attributed to the absorption by plants and the transmission through food chains. Mercury (II) ions (Hg^2+^ ions) in water are concentrated in aquatic organisms and then enriched into the human body, which leads to the dysfunction of cells and damages the central nervous and endocrine systems, consequently giving rise to a series of diseases, including acrodynia disease, Alzheimer’s disease, Hunter–Russell syndrome disease etc. [7,8,9,10]. The tolerable content of inorganic mercury in drinking water is limited according to the World Health Organizations requires. Consequently, rapid and selective recognition of Hg^2+^ ions in vitro and in vivo is essential [11,12,13,14,15].

To date, Hg^2+^ ions could be monitored by several testing technologies such as electrochemical analysis, laser ablations inductively coupled plasma mass spectrometry, atomic absorptions spectrometry, voltammetry, UV–vis spectrometry, and atomic emission spectrometry, etc. [16,17,18]. Compared with these analytical methods, fluorescent techniques have been a promising bio-imaging technique in fields of life sciences, environmental monitoring, and disease diagnosis for their excellent performance targeting metal ions with low cost, good biocompatibility, high sensitivity, selectivity and real-time imaging. Some fluorescent probes have been designed for recognition of Hg^2+^ ions in recent years [19,20,21,22,23,24,25], most of which utilize general fluorophores as signal-output groups such as BODIPY, 1,8-napthalimide, cyanine, coumarin, fluorescein, pyrene, anthracene, and rhodamine etc. [26]. Among a variety of fluorophores, phenylimidazole fluorophore is one of the most famous dyes with a rigid large planar structure and the structure can be flexibly modified. In addition, phenylimidazole has high quantum yield and stable fluorescence and thermodynamic properties under photo-oxidation conditions so that it has a wide application in photochemistry and some probes based on phenylimidazole have been developed [27,28,29,30,31]. Moreover, most of these probes response to Hg^2+^ ions through single emissions intensity change by fluorescence quenching or enhancement, which is likely to be limited to temperature, solvent polarity, medium characteristics, excitations power, dye concentrations, and other factors. Ratiometric fluorescent probes [32,33,34,35,36,37,38,39,40,41] perform through the change of fluorescence intensity at two different emission wavelengths simultaneously. The change of the two emission intensity can efficiently cancel out the interferences of the self-fluorescence and the background fluorescence of the detection system.

**Scheme 1:**
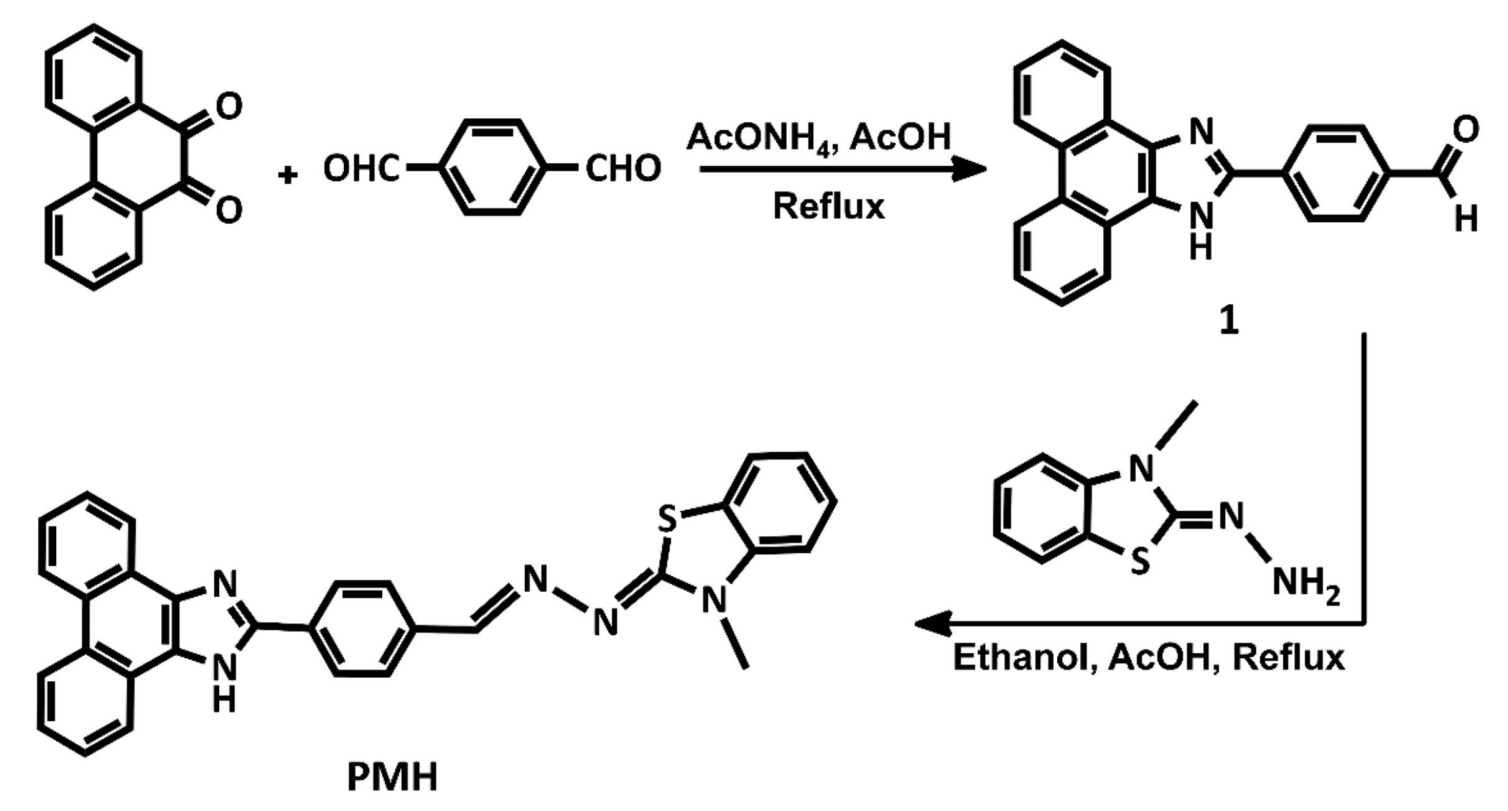
Synthesis procedure of **PMH**.

Herein, a novel ratiometric fluorescence probe **PMH** (((Z)-2-((E)-(4-(1H-phenanthro [9,10-d] Imidazol-2-yl) benzylidene) hydrazono)-3-methyl-2,3-dihydrobenzo [d] thiazole)) for the recognition of Hg^2+^ ions was designed and synthesized based on Schiff base reaction between phenylimidazole fluorophore and 3-methyl-2-benzothiazolinone hydrazone (Scheme 1). The **PMH** probe exhibited a remarkable ratiometric fluorescence response upon the addition of Hg^2+^ ions. The recognition capability of **PMH** to Hg^2+^ ions showed rapid response and excellent stability, which was beneficial to real-time detection. In addition, the **PMH** probe has excellent selectivity towards Hg^2+^ over other metal ions. More importantly, the probe has the potential application in imaging of Hg^2+^ ions in cells and zebrafish.

## 2. Results

### 2.1. Design Strategy of the **PMH** Probe

The **PMH** probe was designed and synthesized in two steps from phenanthrene quinone moiety, which is an important natural product on account of excellent photo-physical properties. Synthesized from phenanthrene quinone, the phenylimidazole moieties have a high fluorescence quantum yield and large stokes shift which is an excellent fluorophore to compose fluorescent probe. Phenylimidazole fluorophore and 3-methyl-2-benzothiazolinone hydrazine were linked together based on Schiff base reaction. The structure of **PMH** was confirmed by ^1^H NMR and ESI-MS. The spectra along could be found in the Supplementary Materials (Figure S1, Figure S2, Figure S3 and Figure S4).

### 2.2. Spectral Titration of **PMH** with Hg^2+^ Ions

**Figure 1:**
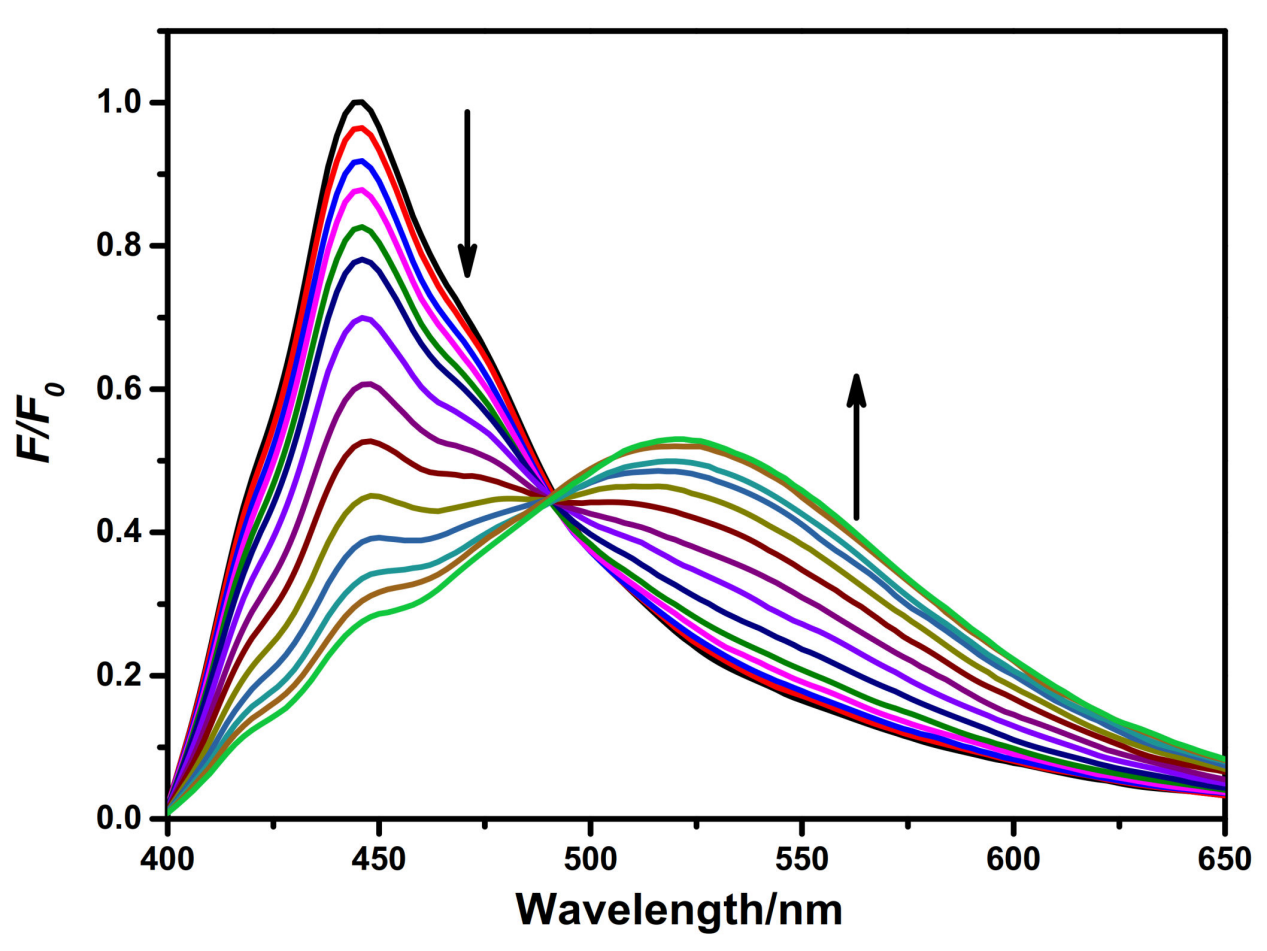
Fluorescence spectra (excited at 380 nm) of **PMH** (20 μM) in the presence of various concentration of Hg^2+^ ions (0–15 equiv.to **PMH**) in acetonitrile/water (8:2, *v*/*v*, 0.1 M KNO_3_, pH = 7.34).

The spectroscopic properties of **PMH** to Hg^2+^ ions were explored by recording the changes of emission spectra in acetonitrile/water (8:2, *v*/*v*, 0.1 M KNO_3_, pH = 7.34) solution. The fluorescence titration of **PMH** in the absence and presence of different amounts of Hg^2+^ ions (0–15 equiv. to **PMH**) was shown in Figure 1. Free **PMH** probe displayed a strong fluorescence emission at 445 nm when excited at 380 nm. After the addition of Hg^2+^ ions, the intensity of the emission peak at 445 nm progressively decreased, and was concomitant with the formation of a new peak around 520 nm. Upon gradual addition of Hg^2+^ ions, the emission intensity at 520 nm increased obviously and tended to be the dominating emission with the addition of mercury (II) ions. The titration curve reached a plateau on the addition of 15 equivalent Hg^2+^ ions. The ratiometric response of the emission peaks before and after treatment with Hg^2+^ ions effectively avoided the emission spectra overlap and the interference of background fluorescence so that it ensured accuracy and high resolution in monitoring of Hg^2+^ ions.

### 2.3. Selectivity and Interference Studies

**Figure 2:**
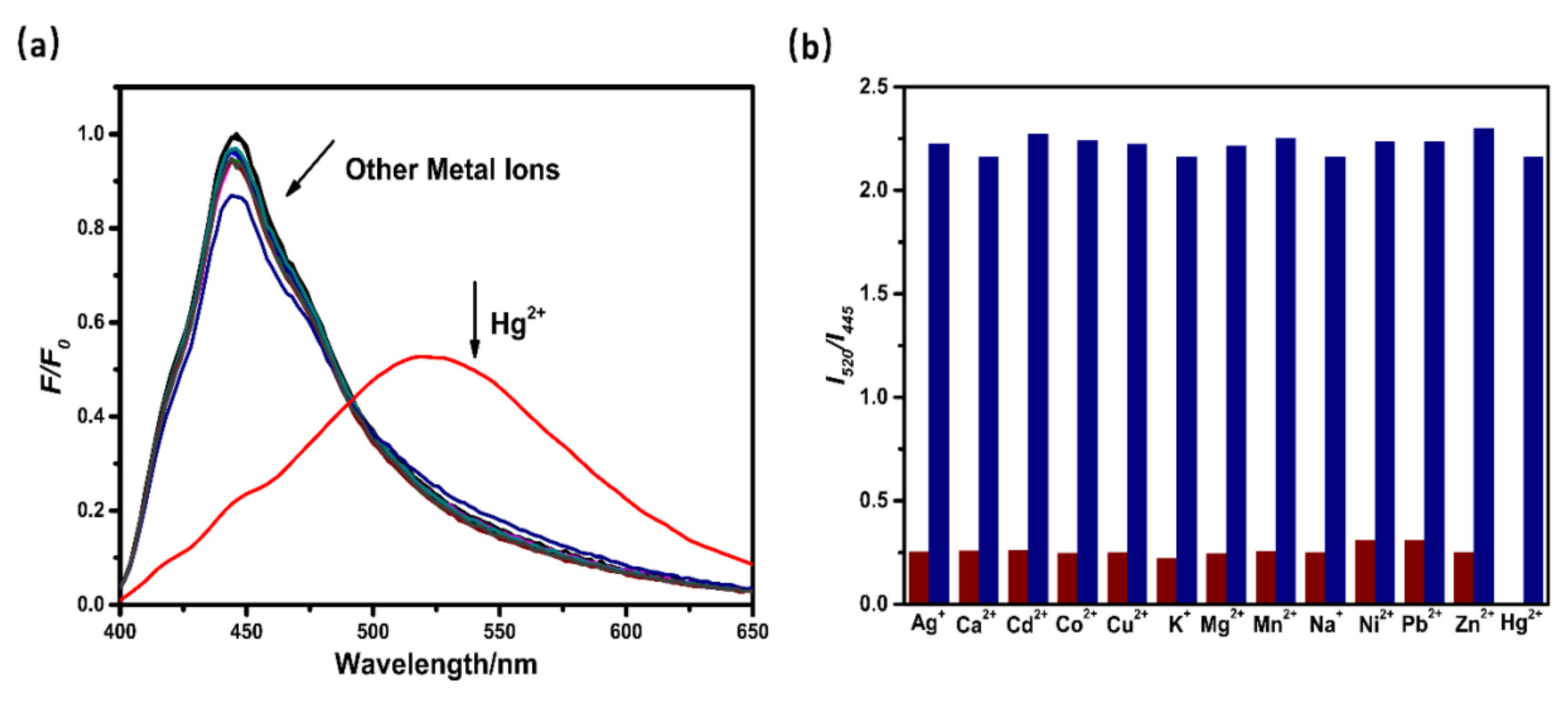
(**a**) Fluorescence response of the **PMH** probe (20 μM) with the addition of various cations (15 equiv. to **PMH**). (**b**) The emission intensity ratio (I_520_/I_445_) of **PMH** (20 μM) to various cations in acetonitrile/water (8:2, v/v, 0.1 M KNO_3_, pH = 7.34) excited at 380 nm. The wine bars in each group represent the emission intensity ratio (I_520_/I_445_) of the **PMH** probe in the presence of 15 equiv. of other cations of interest (Hg^2+^, Ag^+^, Ca^2+^, Cd^2+^, Co^2+^, Cu^2+^, K^+^, Mg^2+^, Mn^2+^, Na^+^, Ni^2+^, Pb^2+^, Zn^2+^). The deongaree bars in each group represent the ratio change that occurred upon subsequent addition of 15 equiv. of Hg^2+^ ions into the above solution.

The selectivity is an important parameter to consider the merits of the probe. To evaluate the selectivity, the fluorescence response of the **PMH** probe for other metal ions was investigated under the solution of acetonitrile/water (8:2, *v*/*v*, 0.1 M KNO_3_, pH = 7.34). As shown in Figure 2a, the addition of other metal ions could not lead to apparent changes of the emission at the wavelength at 445 and 520 nm. In contrast, the addition of Hg^2+^ ions significantly changed the original emission peak mode of the **PMH** probe, which led to obvious quenching at emission of 445 nm and enhancement at the 520 nm. Meanwhile, the fluorescence intensity ratio I_520_/I_445_ would not be influenced by other metal ions (wine bars of Figure 2b). As expected, the ratiometric fluorescent response of **PMH** was highly specific toward mercury ions, and no significant change in fluorescent intensity ratio (I_520_/I_445_) was obtained when the probe was treated with other metal cations. When mercury ions were added to the complex of metal ions and **PMH** respectively, the ratio of fluorescence intensity at 520 nm and 445 nm (I_520_/I_445_) increased instantaneously, indicating that the recognition of Hg^2+^ ions could not be interfered by other metal ions (deongaree bars of Figure 2b). The results show that the **PMH** probe has high selectivity towards Hg^2+^ ions. 

### 2.4. Stability and Time Response

**Figure 3:**
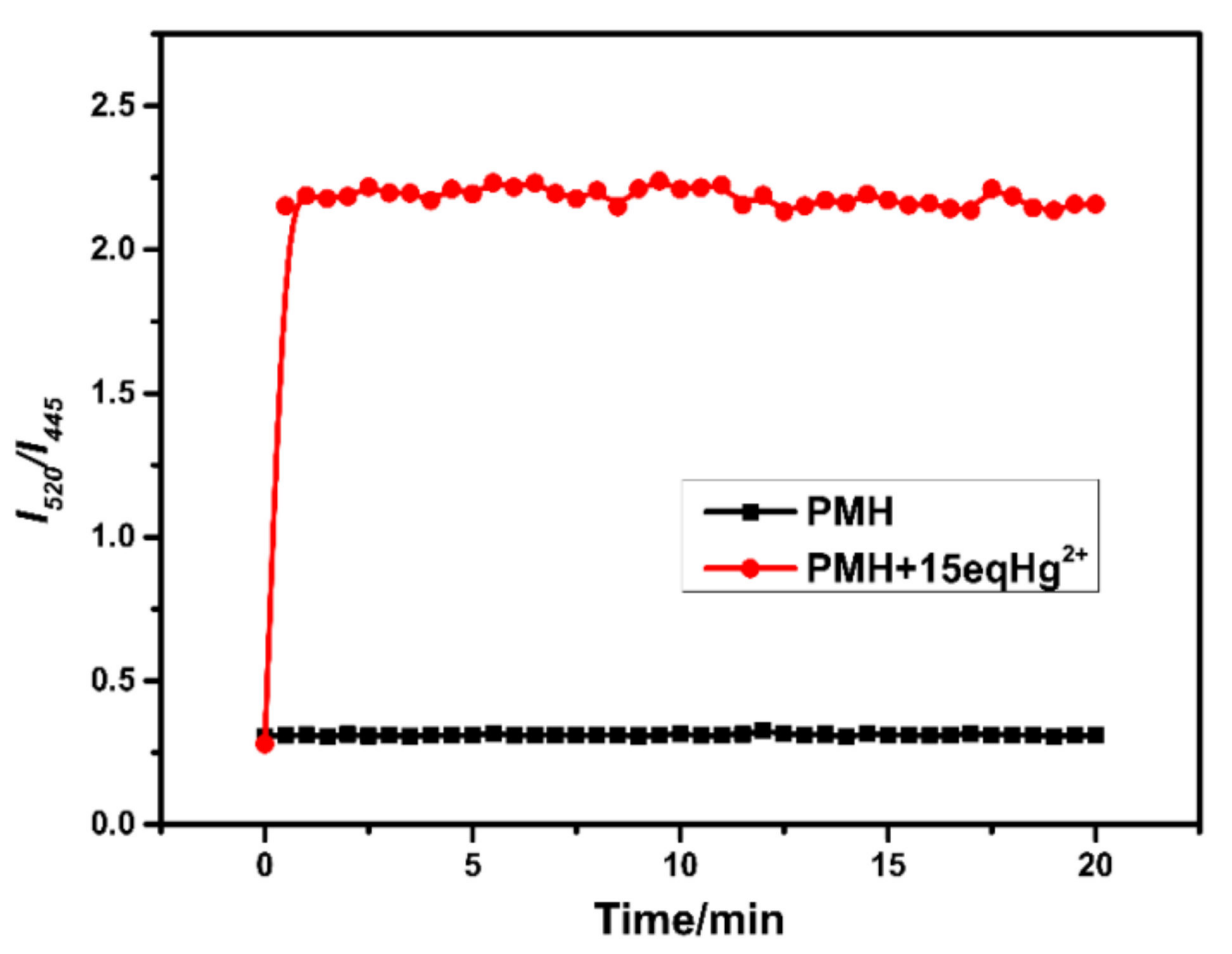
Time-dependent fluorescence intensity ratio (I_520_/I_445_) of the **PMH** probe (black line) and **PMH** with the addition 15 equiv. of Hg^2+^ ions (red line) in solutions of acetonitrile/water (8:2, *v*/*v*, 0.1 M KNO_3_, pH = 7.34) (λ_ex_ = 380 nm).

Stability and response time are important criteria for designing new fluorescence probes. The time-dependent fluorescence experiments were designed and performed. First, the free **PMH** probe was added into solution of acetonitrile/water (8:2, *v*/*v*, 0.1 M KNO_3_, pH = 7.34) and the fluorescence signal of **PMH** was apparently stable within 20 min (shown in Figure 3). Then, upon the additions of 15 equivalent Hg^2+^ ions to the probe, the fluorescence emission intensity ratio (I_520_/I_445_) increased instantaneously and reached the maximum within seconds, the fluorescence signal maintain stable within 20 min as well. All these phenomena indicated that the fluorescence of **PMH** in the absence and presence of Hg^2+^ ions could remain stable for a period of time. The good stability and rapid response to mercury ions of the probe **PMH** is particularly important for the practical real-time detection.

### 2.5. Fluorescence Imaging in Living Cells

**Figure 4:**
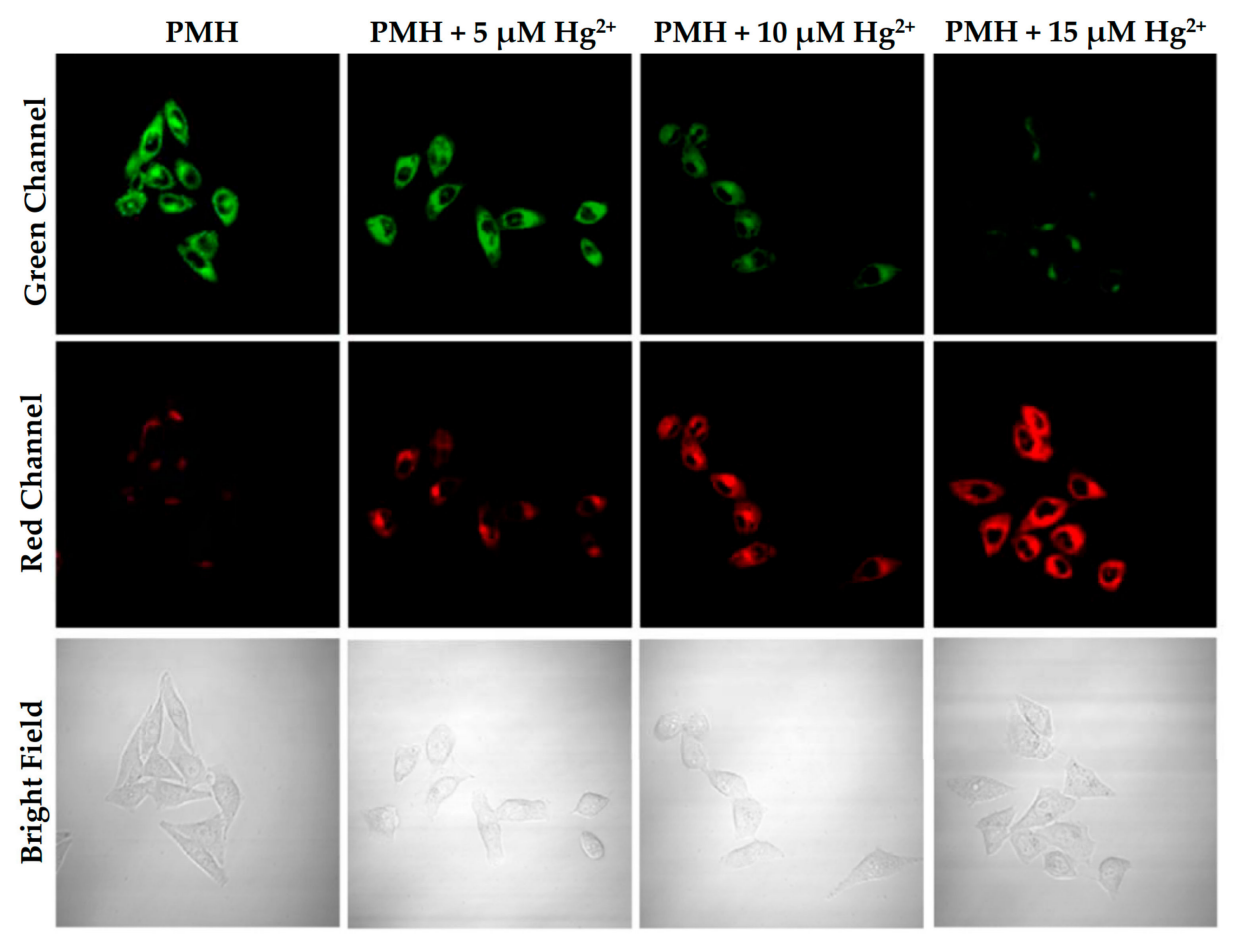
Confocal microscopy images of MCF-7 cells with **PMH** and Hg^2+^ ions (excited at 405 nm).treated with **PMH** (1 μM) and Hg^2+^ ions (0 μM, 5 μM, 10 μM, 15 μM) Green Chanel: fluorescent image of emission between 430–460 nm; Red Channel: fluorescent image of emission between 490–550 nm.

To test the feasibility of the **PMH** probe for sensing cellular Hg^2+^ ions, ratiometric fluorescence imaging of MCF-7, A549, Hela cells by confocal microscope were carried out and the images were presented in Figure 4, Figure S6, and Figure S7. First, MCF-7 cells were incubated with **PMH** (1 μM) and then subjected to confocal laser scanning microscopy. As shown in Figure 4, MCF-7 cells exhibited bright fluorescence in the green channel, but low intracellular fluoresence in the red channel. Upon addition of different concentration of Hg^2+^ ions (0, 5, 10, 15 μM) to the **PMH**-stained cells, fluorescence intensity in the red channel increased dramatically, while the fluorescence in the green channel decreased markedly. Meanwhile, A549 cells and Hela cells were treated with **PMH** (1μM) and different concentration of Hg^2+^ ions (0, 5, 10, 15 μM) respectively. The images were observed under confocal fluorescence microscope and presented in Figure S5 and  Figure S6. Similarly, cells only treated with **PMH** exhibited bright fluorescence in the green channel and weak fluorescence in the red channel. Upon addition of Hg^2+^ ions to the **PMH**-stained cells, fluorescence increased dramatically in the red channel and decreased markedly in the green channel. These results demonstrated that the probe was cell-permeable and suitable for imaging Hg^2+^ ions in different cells lines.

### 2.6. Zebrafish Imaging

**Figure 5:**
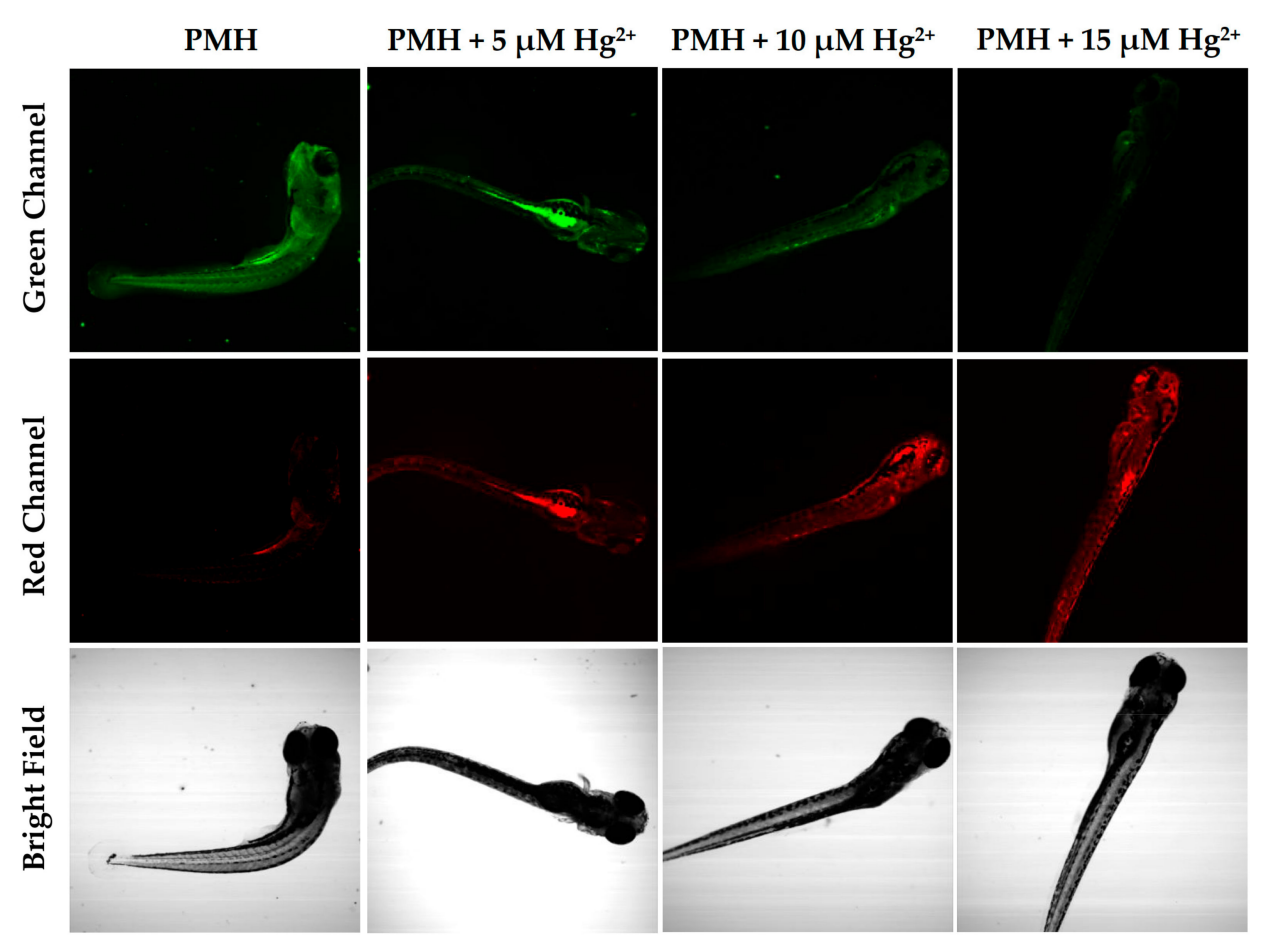
Confocal microscopy images of zebrafish embryos with **PMH** and Hg^2+^ ions. Zebrafish was incubated with the **PMH** probe (1 μM) and followed by different concentration of Hg^2+^ ions (0 μM, 5 μM, 10 μM, 15 μM). λ_ex_ = 405 nm, Green Channel: fluorescent image of emission between 430–460 nm; Red Chanel: fluorescent image of emission between 490–550 nm.

Zebrafish is known as an excellent vertebrate model organism which is widely used in biology. In view of the advantage of rapid development and transparency of the embryos of zebrafish, the visualization of recognition substance and the potential application can be researched by fluorescence imaging. As shown in Figure 5, zebrafish incubated with **PMH** showed obvious fluorescence in the green channel and weak fluorescence in the red channel, indicating that the probe could be easily absorbed and distributed throughout the whole organism. As the concentration of Hg^2+^ ions increased from 0 to 15μM, the fluorescence of the green channel gradually decreased while the fluorescence of the red channel enhanced markedly, exhibiting that **PMH** had excellent imaging feasibility in zebrafish. The results imply that the probe has excellent penetration and is suitable for rapid visualization Hg^2+^ ions zebrafish.

## 3. Experimental

### 3.1. Materials and General Method

9,10-phenanthroquinone, terephthalaldehyde, KNO_3_ were purchased from Energy Chemical (Shanghai, China). 3-methyl-2-benzothiazolinone hydrazone hydrochloride monohydrate and the salts of metal ions (NaClO_4_·H_2_O, Fe(ClO_4_)_2_·6H_2_O, Zn(ClO_4_)_2_·6H_2_O, Cu(ClO_4_)_2_·6H_2_O, KClO_4_, Ca(ClO_4_)_2_·4H_2_O, Mg(ClO_4_)_2_·6H_2_O, AgClO_4_·6H_2_O, Mn(ClO_4_)_2_·6H_2_O, Cd(ClO_4_)_2_·6H_2_O, Co(ClO_4_)_2_·6H_2_O, Ni(ClO_4_)_2_·6H_2_O, Pd(ClO_4_)_2_·6H_2_O,Hg(ClO_4_)_2_·3H_2_O) were purchased from J&K Scientific (Shanghai, China). Solvents such as glacial acetic acid, methanol, ethanol, acetonitrile are commercially available analytical grades and were used directly. Experimental water was deionized water. ^1^H NMR spectra were measured on a Bruker AVANCE III 500 (Bruker, Bremen, Germany) at room temperature. Mass spectra were carried out on a LTQ Orbitrap XL spectrometer (Thermo Scientific, MA, USA) using methanol as mobile phase. The reactions were monitored by TLC and were visualized by UV lamp (254 nm and 365 nm). The fluorescent spectra were measured on EDINBURGH FS920 (Edinburgh Instruments, UK), Δλ = 1.1 nm with the excitations at 380 nm. Fluorescence images of cells were recorded by an Olympus FV1000 confocal microscope (Olympus, Kyoto, Japan).

### 3.2. Synthesis of Compound **1** (4-(1H-phenanthro[9,10-d]imidazol-2-yl)benzaldehyde)

A mixture of 9,10-phenanthroquinone (208 mg, 1 mmol), ammonium acetate (153.9 mg, 2 mmol), and terephthalaldehyde (135.2 mg, 1 mmol) were stirred in glacial AcOH (15 mL) under reflux conditions for 30 min [42]. The hot solution was cooled to room temperature, and the resulting yellow solid was collected by filtrations and washed with an excess amount of water and methanol to remove starting materials. Yield: 260 mg, 79.43%. ^1^H NMR (500 MHz, DMSO-*d_6_*) δ 13.66 (s, 1H), 10.05 (s, 1H), 8.81 (m, 2H), 8.62 (d, 1H), 8.56 (d, 1H), 8.50 (d, 2H), 8.08 (d, 2H), 7.74 (m, 2H), 7.63 (m, 2H). ESI-MS: *m*/*z* = 323.1176 for [M + H]^+^.

### 3.3. Synthesis of **PMH** (((Z)-2-((E)-(4-(1H-phenanthro[9,10-d]imidazol-2-yl) benzylidene) hydrazono)-3-methyl-2,3-dihydrobenzo[d]thiazole))

Compound **1** (322 mg, 1 mmol) was dissolved in 20 mL of hot absolute ethanol, then 3-methyl-2-benzothiazolinone hydrazone hydrochloride monohydrate (233 mg, 1 mmol) was added to the solution. The mixture was stirred under reflux conditions for 4 h to attain the product [43]. The yellow precipitate was filtrated and washed with hot absolute ethanol for three times and dried in vacuum to give compound **PMH.** Yield: 432 mg, 78.21%.^1^H NMR (500 MHz, DMSO-*d_6_*) δ 13.76 (br, 1H), 8.87 (t, 2H), 8.69 (m, 2H), 8.62 (s, 1H), 8.48 (d, 2H), 7.97 (d, 1H), 7.75 (m, 3H), 7.67 (m, 3H), 7.34 (m, 2H), 7.13 (t, 1H), 3.61 (s, 3H). ESI-MS: *m*/*z* = 484.1580 for [M + H]^+^.

### 3.4. Spectrophotometric Experiments

The salts of metal ions were dissolved in distilled water to afford 1.0 × 10^−2^ M mother liquors. **PMH** was dissolved in dimethyl sulfoxide to prepare a solution with the concentrations of 1.0 × 10^−3^ M. Aliquots of stock solutions of **PMH** were diluted to 2 mL acetonitrile/water (8:2, *v*/*v*, 0.1M KNO_3_, pH = 7.34) to make the final concentrations of 20 μM. The selective assay of **PMH** (20 μM) was researched in the presence of various competitive species under the same condition were recorded by I_520_/I_445_ excited at 380 nm.

### 3.5. Cell Culture and Imaging

MCF-7, Hela cell lines were cultured in Dulbecco’s modified Eagle’s medium (DMEM) and A549 cells were cultured in RPMI-1640 including 10% fetal bovine serum at 37 °C in an incubator containing 5% CO_2_ respectively. For imaging of Hg^2+^ in living cells, the cells were treated with 1 μM of **PMH** under the growth medium and then incubated with varied concentrations of Hg^2+^ ions (0, 5, 10, 15 μM). The cells were washed by PBS three times and then cell images were obtained via a confocal microscope from Olympus FV1000 laser confocal microscope at excitations of 405 nm. The green channel collected the emission wavelength between 430–460 nm and the red channel collected the emission wavelength between 490–550 nm.

### 3.6. Zebrafish Imaging

Zebrafish embryos were maintained at 28 °C and washed with E3 for fluorescence imaging. For ratiometric imaging, the green channel collected the emission wavelength between 430–460 nm and the red channel collected the emission wavelength between 490–550 nm. Confocal fluorescence images were recorded on an Olympus FV1000 laser confocal microscope at excitation of 405 nm.

## 4. Conclusions

In conclusion, a novel phenylimidazole-based ratiometric fluorescent probe **PMH** has been developed and synthesized by Schiff base reaction. **PMH** probe could recognize Hg^2+^ rapidly and present excellent stability. The addition of Hg^2+^ ions to **PMH** results in a ratiometric fluorescence response with a dramatic enhancement of fluorescence intensity at 520 nm and a significant decrease at 445 nm. **PMH** shows high selectivity toward Hg^2+^ ions over other metal ions. Remarkably, the probe has excellent biocompatible and successful utilization in bio-imaging in cells and zebrafish.

## Supplementary Materials

Supplementary data associated with this article can be found in the online.
