# Supplementary material for: A Novel Ratiometric Fluorescent Probe for Mercury (II) ions and Application in Bio-imaging
Source: Molecules. 2019 Jun 18;24(12):2268. doi: 10.3390/molecules24122268 (PMC6630643; doi:10.3390/molecules24122268)
Supplement: Supplementary file 1 [file molecules-24-02268-s001.pdf]

Supplementary Information

A Novel Ratiometric Fluorescent Probe for Mercury (II) ions and Application in Bio-imaging

Qianmiao Gao, [1] **Yang Jiao,** [1,2,*] **Cheng He,**[1] **Chunying Duan**[1]

1. State Key Laboratory of Fine Chemicals, Dalian University of Technology, Dalian 116024, China;

2. School of Chemical Engineering, Dalian University of Technology, Dalian 116024, China

E-mail: jiaoyang@dlut.edu.cn

Figure S1. ^{1}H NMR of compound 1

Figure S2. ESI-MS spectrum of compound 1

Figure S3. ^{1}H NMR of PMH

Figure S4. ESI-MS spectrum of PMH

Figure S5. Confocal microscopy images of A549 with PMH and Hg^{2+} ions

Figure S6. Confocal microscopy images of Hela with PMH and Hg^{2+} ions

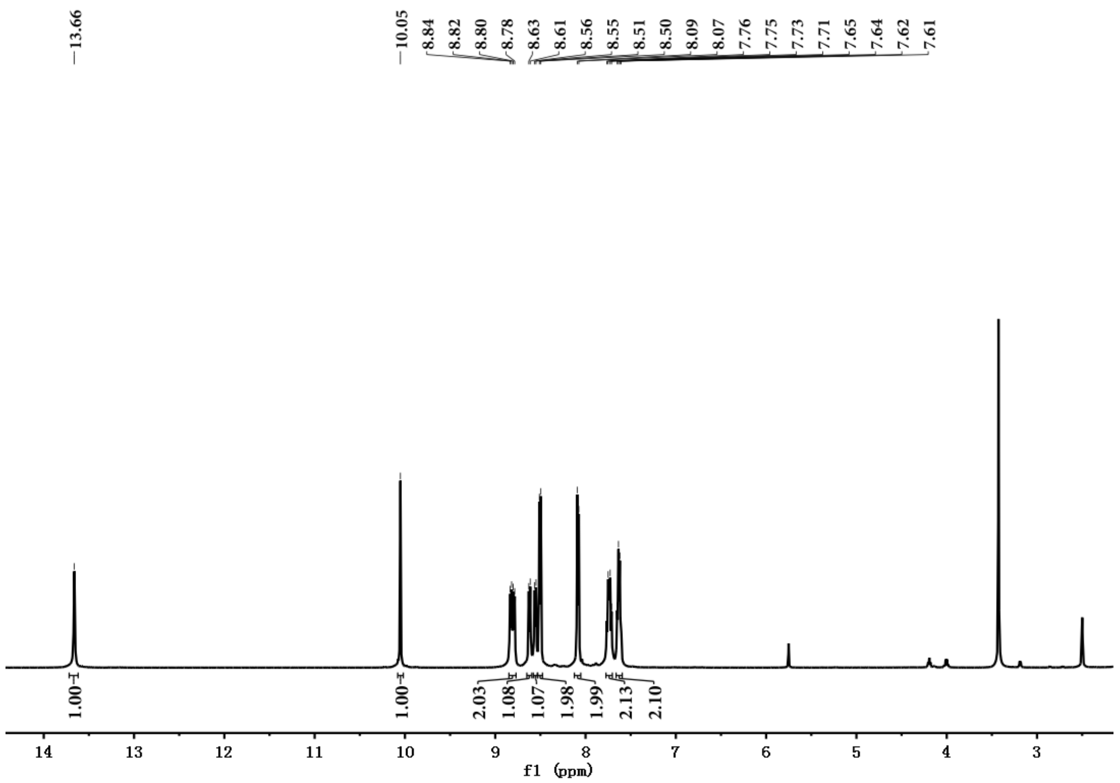

Figure S1. ^{1}H NMR of compound **1**

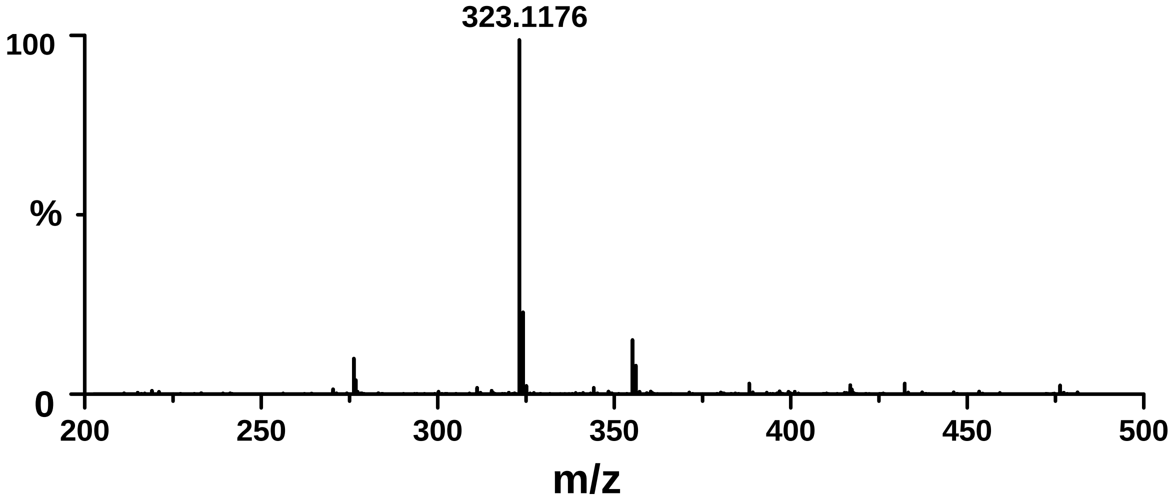

Figure S2. ESI-MS spectrum of compound **1**.

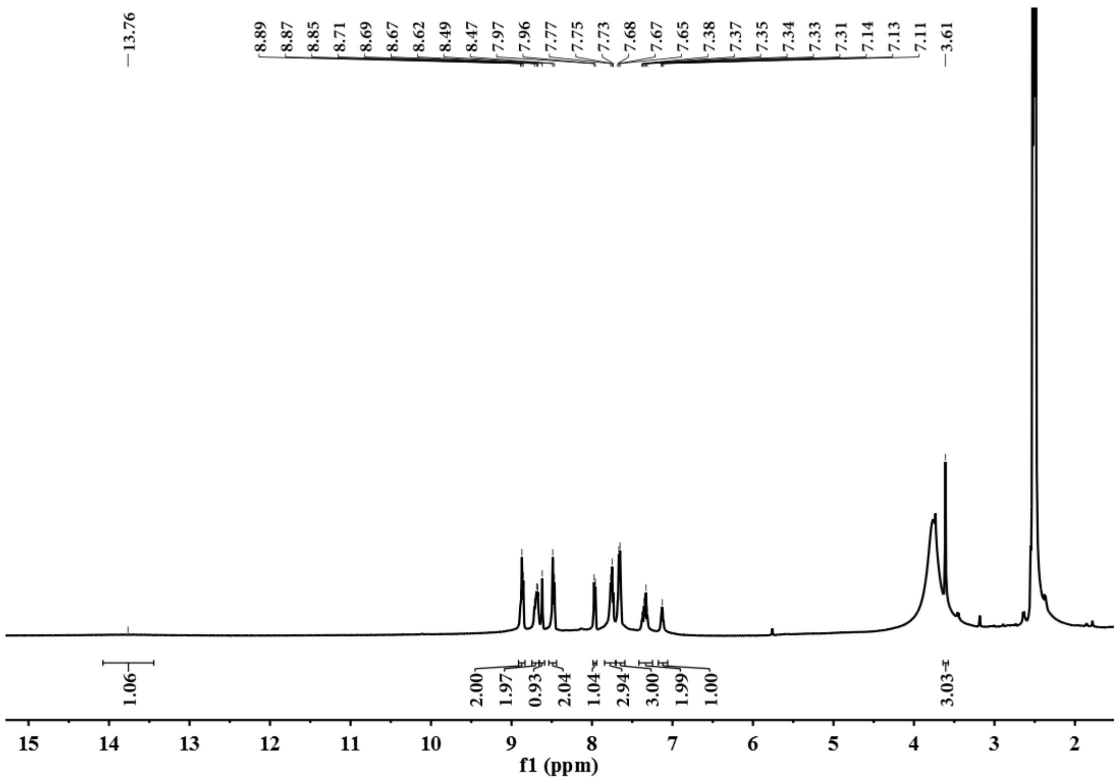

Figure S3. ^{1}H NMR of **PMH**.

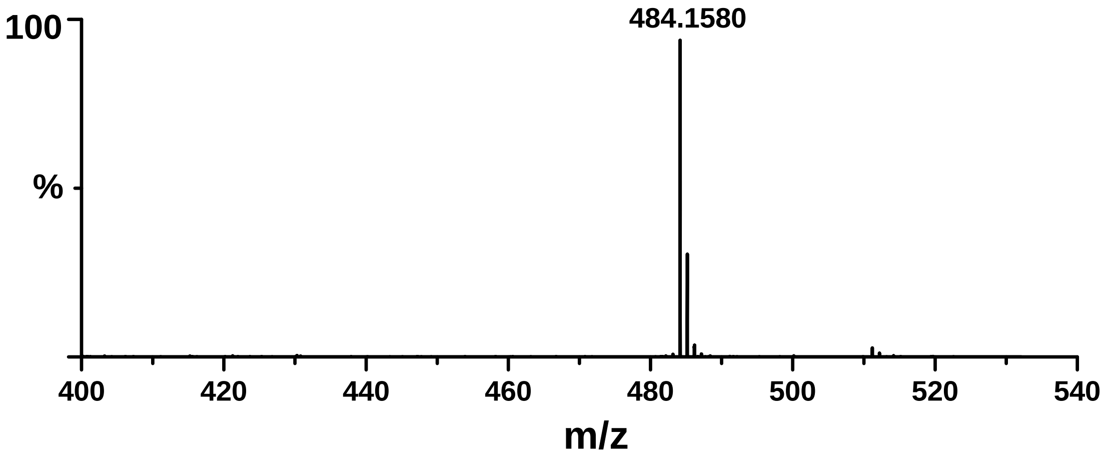

Figure S4. ESI-MS spectrum of **PMH**.

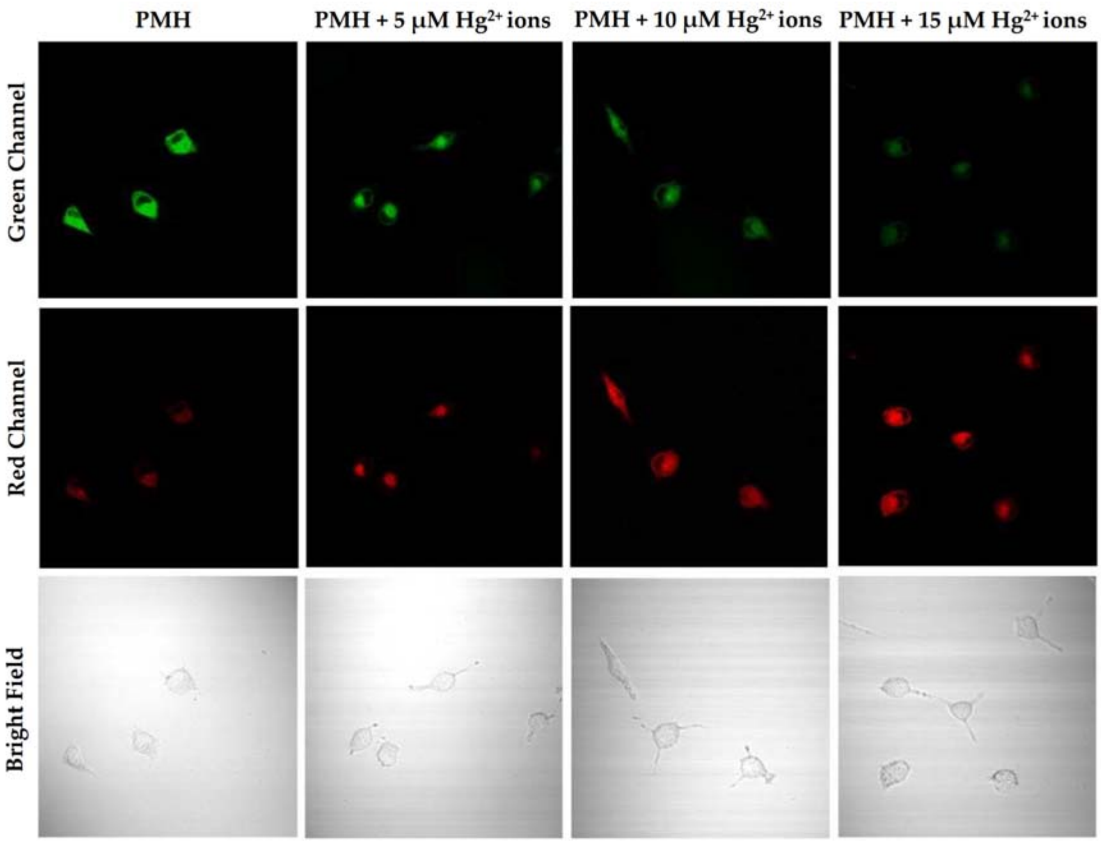

Figure S5. Confocal microscopy images of A549 cells treated with **PMH** (1 μM) and different concentration of Hg^{2+} ions (0 μM, 5 μM, 10 μM, 15 μM) (excited at 405 nm), Green Chanel: fluorescent image of emission between 430–460 nm; Red Channel: fluorescent image of emission between 490–550 nm.

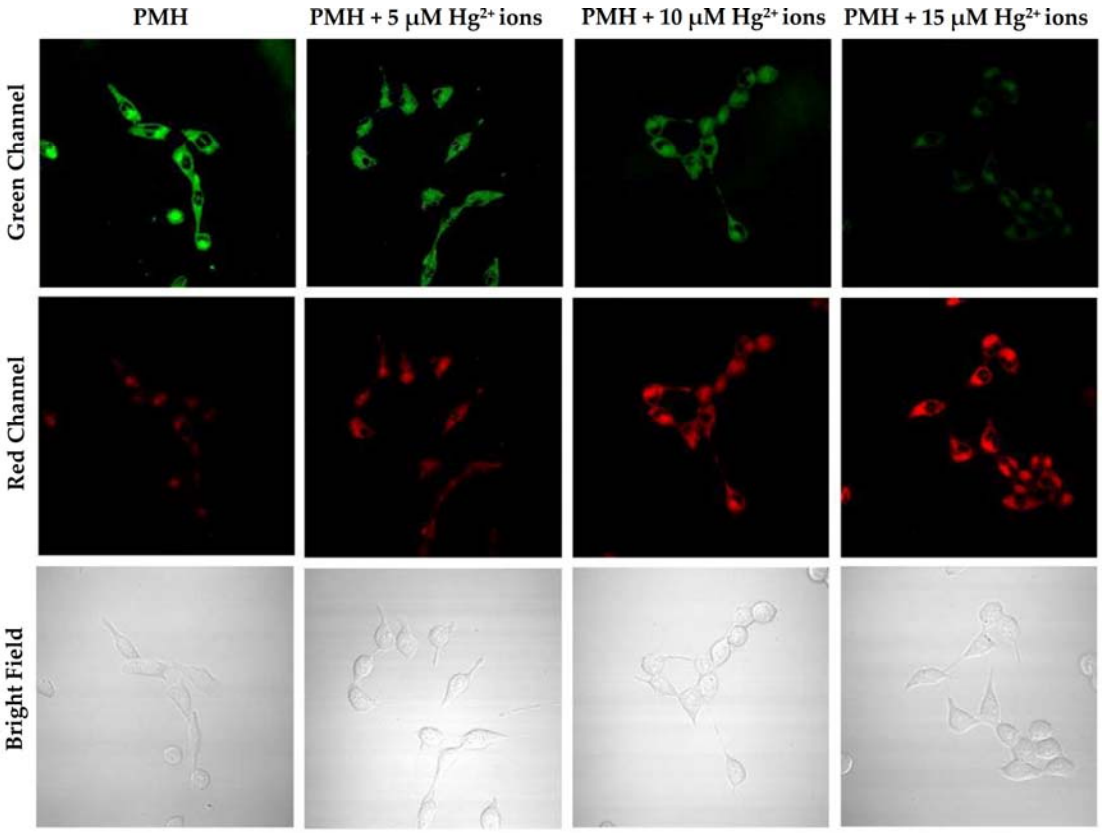

Figure S6. Confocal microscopy images of Hela cells treated with **PMH** (1 μM) and different concentration of Hg^{2+} ions (0 μM, 5 μM, 10 μM, 15 μM) (excited at 405 nm), Green Chanel: fluorescent image of emission between 430–460 nm; Red Channel: fluorescent image of emission between 490–550 nm.
